# Quality of life and patient safety: the impact of the work environment on the well-being of medical staff in ICU settings

**DOI:** 10.62838/jccm-2026-0025

**Published:** 2026-07-27

**Authors:** Sebastian Isac, Elisabeta Nita, Victor Cristian Toma, Andrada-Georgiana Badea, Mihaela Camelia Popa, Teodora Isac, Mara Madalina Mihai, Cristina Veronica Andreescu, Cristina Martac, Gabriela Droc

**Affiliations:** Department of Anesthesiology and Intensive Care I, Fundeni Clinical Institute, Bucharest, Romania; Institute of Philosophy and Psychology “Constantin Radulescu-Motru”, Romanian Academy, Bucharest, Romania; Department of Internal Medicine II, The Faculty of Medicine, Carol Davila University of Medicine and Pharmacy, Bucharest, Romania; Department of Oncologic Dermatology and Allergology, Carol Davila University of Medicine and Pharmacy, Bucharest, Romania; Department of Foreign Languages, Carol Davila University of Medicine and Pharmacy, Bucharest, Romania

**Keywords:** burnout, compassion satisfaction, patient safety, medical staff, intensive care unit

## Abstract

**Background and aim:**

Professional Quality of Life (ProQoL) is essential for understanding the well-being of the health-care professionals working in high-stress environments, such as the intensive care units (ICUs). - This study aimed to assess the influence of age, gender, hospital affiliation, and professional role on the ProQoL dimensions (Compassion Satisfaction – CS, Burnout – BO, Secondary Traumatic Stress – STS) and to analyze the relationship between the perceptions of patient safety (measured via the Safety Attitudes Questionnaire – SAQ) and the professional quality of life.

**Method:**

This cross-sectional observational study included 247 healthcare professionals (April–November 2024) from 20 different hospitals, working in ICUs (nurses, attending physicians, medical residents -, and other staff). Participants filled out the ProQoL and SAQ questionnaires out of which only 10 SAQ items were used. Statistical analyses were performed using ANOVA, t-tests, and Pearson correlation coefficients.

**Results:**

Medical residents and participants in the 20–30 age group reported having the highest burnout scores (28.37 ± 0.37), and the lowest level of compassion satisfaction score (36.65±0.61), while attending physicians had elevated levels of secondary traumatic stress (25.69±0.57). Positive perceptions of the work environment (safety, conflict resolution, workplace satisfaction) were negatively correlated with the burnout (r = −0.5888, p < 0.0001). Finally, the perception of a pleasant job and workplace positively correlates with the CS score (r=0.53 p˂0.0001).

**Conclusions:**

Professional well-being varies significantly in terms of age, and position. These findings suggest that workplace strategies focusing on safety, teamwork, and workload balance may play a role in supporting the ICU staff well-being.

## Introduction

Professional Quality of Life (ProQoL) is a key concept in understanding the emotional and psychological well-being of healthcare workers, who are frequently exposed to high levels of stress and traumatic events in the workplace [1]. The ProQoL instrument, developed by Stamm [2], is widely used to measure both the positive and negative effects of caregiving, including the job satisfaction and the emotional strain. It measures three decisive dimensions: Compassion Satisfaction (CS), Burnout (BO), and Secondary Traumatic Stress (STS).

The concept of Compassion Fatigue (CF), which includes the burnout and the secondary traumatic stress, reflects on the negative consequences of working with people having undergone trauma or violence. In terms of symptoms, it may manifest in in a manner resembling the post-traumatic stress disorder (PTSD), thus impacting the multiple domains of the functional capability of the caregiver [3]. Although a number of studies have highlighted the relevance of the compassionate satisfaction as a protective factor against compassion fatigue [4], the construct validity of the ProQoL instrument remains a subject of debate.

The COVID-19 pandemic has called attention to the importance of assessing and improving the quality of work life, especially for the healthcare workers directly involved in the care of critically ill patients [5]. Studies report moderate levels of burnout and STS, along with sustained compassion satisfaction among the ICU workers at different workloads [6,7]. Positive work environments and targeted psychological support have been associated with lower risks of compassion fatigue and improved well-being [8,9].

Although numerous studies have documented the effectiveness of interventions to reduce the compassion fatigue [10], the methodological heterogeneity and the risk of bias emphasize the need for further research to optimize the intervention strategies [11]. ProQoL remains a valuable tool for examining the emotional costs and rewards of the caregiving professions.

Assessing the perceptions of safety of the healthcare professionals is essential for developing a strong safety culture. The Safety Attitudes Questionnaire (SAQ) has been attested to be a validated, widely used instrument to measure distinct dimensions such as team climate, job satisfaction, and management perception.

Recent international studies underline the usefulness of the SAQ in various contexts: by improving safety scores through training in Malaysia [12], by adapting it for primary care in Denmark [13], or by evaluating it in diverse cultural contexts, in particular Ethiopia and Iran [14],[15]. Such findings support the value of the SAQ in fostering a strong safety culture in healthcare.

Although ProQoL and SAQ have been widely used independently, few studies have investigated the association between professional well-being and perceived patient safety across multiple professional categories in ICU settings.

The primary objective of this study was to assess how the influence of age, gender, hospital type, and professional role affect the three core ProQoL components. The secondary objective included comparing the SAQ item responses across the provider groups and examining their correlations with ProQoL scores in the ICU settings.

## Materials and methods

This research consists in a multicentric observational study that includes professional healthcare providers with the main activity in the ICU settings from 20 national hospitals (7 university-affiliated hospitals and 13 non-university affiliated hospitals). The study was conducted between April and November 2024 and involved and included 247 participants (182 females, 65 males). Informed consent was obtained prior to participant enrollment. The procedures used in this study adhere to the tenets of the Declaration of Helsinki. Ethical approval was obtained from the Ethics Committee of Fundeni Clinical Institute, Bucharest, Romania (1032/02.04.2024). The ICU departments were mainly mixed departments consisting in surgical and medical critical patients and having a patient’s capacity between 10 and 24.

This study specifically examined the intersection between professional quality of life and patient safety climate perceptions among intensive care unit staff, using the Professional Quality of Life (ProQoL) scale and the Safety Attitudes Questionnaire (SAQ).

The participants were asked to provide answers to a survey consisting of 30 items included in the ProQoL Questionnaire (Romanian validated version) and other 10 items included in the SAQ questionnaire (Romanian validated version). The structure of the questionnaires is revealed in supplementary file. The BO and STS scores received points from 1 to 30 and the CS score from 1 to 50. We selected 10 items from the validated SAQ tool based on their relevance to ICU work climate, collaboration, and safety perception. The rationale was to minimize response fatigue while preserving psychometric robustness. For the 10 items included in the patient safety perception among various healthcare providers categories, the scores varied from 1 to 6 per each item, where 1 represented strong disagreement and 6 strong agreements, respectively. No formal, standardized BO prevention programs were in place during the study period in any of the participating units.

To evaluate the influence of the age, gender, category of the healthcare provider and the status of the hospital on the ProQoL score, we performed a comparative analysis, dividing the patients into various subgroups and compared the mean scores divided into CS score, BO score, and ST. Regarding the age, we divided the participants in 4 age intervals: the 20–30 y.o. (n= 66), the 31–40 y.o. (n=74), the 41–50 y.o. (n=77) and the over 50 y.o. (n=30). In terms of gender, we split the responders into two groups: male (n=65), and female (n=182). Considering the category of the healthcare provider, the participants were separated into four groups: nurses (N) (n=85), medical residents (MR) (n=71), medical doctors (MD) (n=72), and others (O) (n=19). We considered the other healthcare provider the personnel who are not nurses, resident doctors, or attending physicians like registrars, secretary staff etc. Finally, we considered the hospital status of the responders as university-affiliated (n=177) and non-university-affiliated (n=70).

To perform the comparative analysis of the mean scores of the items included in the SAQ questionnaire in reference to the category of the healthcare provider, we resorted to the four subgroups above-mentioned: nurses (N) (n=85), medical residents (MR) (n=71), medical doctors (MD) (n=72), and others (O) (n=19).

The data were analyzed via the GraphPad Prism 6.00 (GraphPad Software Inc.). One-way ANOVA followed by post hoc Tukey’s test for interactions between groups was employed for the comparative analysis, in which more than three groups were included and the t-test for comparative analysis between two groups, respectively. The data are expressed as the mean ± SEM. A two-sided P value <0.05 was considered statistically significant.

For the correlative analysis, there were considered as independent variables the items included in the SAQ questionnaire and the dependent variables, the three scores from the ProQoL: CS, BO, STS. The Pearson correlation coefficient, r, was determined. A two-sided P value <0.05 to indicate statistical significance for each correlation was considered. The r values under 0.4 were assigned to have negligible correlative effects. Finally, the nonsignificant correlations were excluded.

## Results

**Fig.1:**
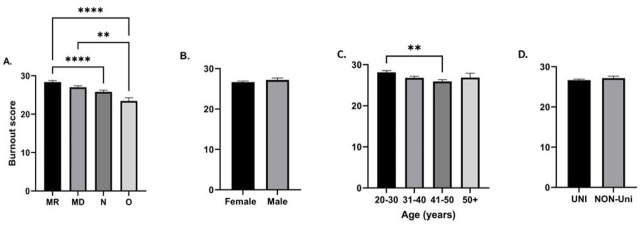
**Differences in burnout score with regard to the category of the health care provider (A), gender (B), age (C), and the hospital status (D). The date is illustrated as means±SEM.** ** represents a p value between 0.001–0.01, **** represents a p value less than 0.0001.

**Fig.2:**
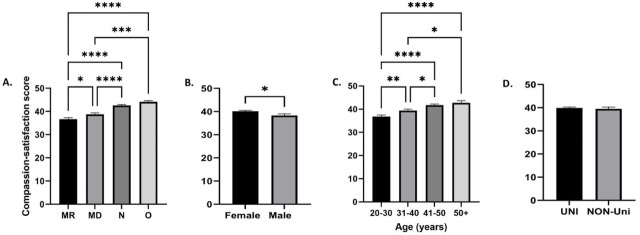
**Differences in compassion-satisfaction score with regard to the category of the health care provider (A), gender (B), age (C), and the hospital status (D).** The date is illustrated as means±SEM. * represents a p value between 0.05–0.01, ** represents a p value between 0.001–0.01, *** represents a p value between 0.0001–0.001, and **** represents a p value less than 0.0001.

**Fig.3:**
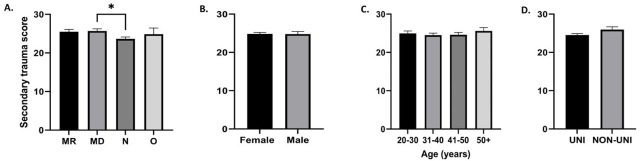
**Differences in the secondary trauma score with regard to the category of the health care provider (A), gender (B), age (C), and the hospital status (D).** The date is illustrated as means±SEM. * represents a p value between 0.05–0.01.

The comparative analysis of the mean scores from the ProQoL questionnaire with regard to the age, gender, category of the healthcare provider and the status of the hospital are revealed in the figures 1–3.

Figure 1 reveals the means of the BO score considering the category of the healthcare provider (Fig.1A), gender (Figure 1B), age (Fig.1C), and the status of the hospital (Figure 1D). We observed a significantly higher BO score in the MR group (28.37 ± 0.37) compared to the O group (23.43 ± 0.83) (p < 0.0001), the MR group (28.37±0.37) vs. the N group (25.81±0.43) (p < 0.0001), and between the MD group (27.01±0.4) and the O group (23.43±0.83) (p=0.004) (Figure 1A). No differences were observed in the BO scores with respect to gender (Female 26.66±0.27 vs. Male 27.18±0.5, p=NS) (Fig.1B), or to the status of the hospital (UNI 26.66±0.27 vs. 27.17±0.49, p=NS) (Figure 1D). On the subject of the age of the responders, we revealed that the youngest responders tend to have the highest BO score. The only significant difference was noticed, however, between the 20–30 y.o. group-28.14±0.41-the highest B.O. score and the 41–50 y.o. group-25.91±0.44, the lowest B.O. score p=0.002 (Fig.1C).

Figure 2 reveals the means of the CS score, considering the healthcare provider (Fig.2A), gender (Figure 2B), age (Fig.2C), and the status of the hospital (Figure 2D). In reference to the healthcare provider category, we drew attention to the lowest CS score in the MR group (36.65±0.61), which was statistically significant when compared to the other three groups: the MD group (38.74±0.59, p=0.041), the N group (42.54±0.44, p < 0.0001), and the O group (44.14±0.51, p < 0.0001) (Figure 2A). Furthermore, the CS score in the MD (38.74±0.59, p=0.041) group was significantly decreased when compared to the N (42.54±0.44, p < 0.0001), and the O (44.14±0.51, p < 0.0001) groups (Figure 2A).

Additionally, the women showed a higher CS score, when compared to men: 40.15±0.38 vs. 38.32±0.71 (p=0.03) (Fig.2B). Considering the age of the responders, we remarked the lowest CS score in the 20–30 y.o. group (36.79±0.68), which was statistically significant, when compared to all other groups: the 31–40 y.o. (39.45±0.63, p=0.009), the 41–50 y.o. (41.79±0.43, p < 0.0001), and the 50+ y.o. (42.71±0.98, p < 0.0001) (Figure 2C). Furthermore, the 31–40 y.o. group exhibited lower CS score, when compared to the 41–50 y.o., and the 50+ y.o. groups: 39.45±0.63 vs. 41.79±0.43, p=0.016, and 39.45±0.63 vs. 42.71±0.98, p=0.04, respectively (Fig.2C).

Finally, no significant difference in the CS score was revealed, considering the status of the hospital: 39.90±0.38 (UNI group) vs. 39.54±0.71 (non-UNI group), p=NS (Figure 2D).

The dynamic of the STS score related to the category of the healthcare provider, gender, age, and the status of the hospital is illustrated in the Fig.3A–D. A statistically significant increase of the ST score in the MD group, when compared to the N group: 25.69±0.57 vs. 23.63±0.50, p=0.04 (Figure 3A) was observed.

No further statistically significant differences were noticed in the STS score, considering the gender, and the age of the responders, or the status of the hospital (Figure 3B–D). Among women, the STS score was 24.84±0.36, similar to men- 24.80±0.68, p=NS. The STS score was the same among various age intervals: 24.95±0.66 (the 20–30 y.o. group), 24.50±0.52 (the 31–40 y.o. group), 24.61±0.61 (the 41–50 y.o. group), and 25.61±0.86 (the 50+ y.o. group), respectively. Finally, no statistical significance was achieved with regard to the status of the hospital: 24.55±0.35 (the UNI group) vs. 25.96±0.73 (the non-UNI group), p=NS.

Table 1 reveals the main differences in the patient safety perceptions among various healthcare providers. Nevertheless, a disagreement among the responders, regardless of their professional status, was obvious on the following topics: enough staff for patients and staff often ignore rules (mean scores lower then 3). Conversely, the responders strongly agreed on topics like: safe environment, pleasant perception of the job, and good place to work, respectively (the mean scores generally over 4). The subgroup analysis revealed that nurses disagreed the most on the topic regarding the number of staff members for patient management. On the matter regarding the adequate equipment, the others group returned the strongest agreement, when compared to medical residents, nurses, and medical doctors. The same trend was noted on the topic related to the conflicts solving for the patients’ good.

**Table 1.** Differences in perception of various items included in the Safety Attitudes Questionnaire (SAQ) among medical staff categories.

	**Medical residents (MR) (mean+/− SEM)**	**Nurses (N) (mean+/− SEM)**	**Medical doctors (MD) (mean+/− SEM)**	**Others (O) (mean+/−SEM)**	**ANOVA results (p value)**
Enough staff for patients	2.63+/−0.11	2.22+/−0.12	2.8+/−0.12	2.85+/−0.32	p=0.005
Adequate equipment	3.423+/−0.11	4.078+/−0.10	3.875+/−0.10	4.714+/−0.22	p˂0.0001
Conflicts resolved for patients’ good	3.479+/−0.11	3.93+/−0.09	3.81+/−0.11	4.42+/−0.20	P=0.001
Good teamwork	3.845+/−0.10	3.967+/−0.10	4.278+/−0.07	4.429+/−0.20	P=0.007
Safe environment	4.014+/−0.10	4.156+/−0.11	4.125+/−0.08	4.571+/−0.20	P=0.24
Staff often ignore rules	2.042+/−0.07	1.989+/−0.09	2.194+/−0.09	1.500+/−0.25	P=0.03
Ineffectiveness when tired	3.930+/−0.10	2.600+/−0.09	3.431+/−0.11	2.500+/−0.32	P˂0.0001
Excessive workload hurts performance	3.930±0.10	2.789±0.12	3.472±0.11	2.571±0.35	p˂0.0001
I like my job	3.845±0.09	4.311±0.10	4.083±0.09	4.571±0.29	P=0.02
Good workplace	3.648±0.10	4.244±0.10	4.069±0.08	4.714±0.12	p˂0.001

We observed the highest scores on the topic related to good teamwork in the medical doctors and others groups. All responders, regardless of their professional status agreed that their work is accomplished in a safe environment (p=0.24). The strongest disagreement on the matter concerning ignoring the rules was noticed in the nurse and others groups. The perception on the ineffectiveness when tired was highlighted in the medical residents and medical doctors’ groups, while the medical residents agreed that excessive workload hurts performance. The nurses and other groups liked the most their job and found it to be a good workplace.

In Table 2, the main correlations between various patient safety perception variables and the main 3 scores included in the ProQoL questionnaire were pointed out. The highlighted values represent clinically relevant correlations between them.

**Table 2.** Correlations in the perception of various items included in the Safety Attitudes Questionnaire (SAQ) and burnout, compassion-satisfaction, and secondary trauma scores respectively. r represents the correlation coefficient.

	**CO (r, p)**	**BO (r, p)**	**ST (r, p)**
Enough staff for patients	−0.02917 (0.64)	−0.1472 (0.02)	−0.05600 (0.38)
Adequate equipment	0.2646 (˂0.0001)	−0.3718 (˂0.0001)	−0.1284 (0.04)
Conflicts resolved for patients’ good	0.3217 (˂0.0001)	−0.4247 (˂0.0001)	−0.2443 (0.0001)
Good teamwork	0.2587 (˂0.0001)	−0.3836 (˂0.0001)	−0.1233 (0.052)
Safe environment	0.3270 (˂0.0001)	−0.4777 (˂0.0001)	−0.2098 (0.0009)
Staff often ignore rules	−0.3231 (˂0.0001)	0.3865 (˂0.0001)	0.2364 (0.0002)
Ineffectiveness when tired	−0.4704 (˂0.0001)	0.5727 (˂0.0001)	0.3846 (˂0.0001)
Excessive workload hurts performance	−0.4124 (˂0.0001)	0.5186 (˂0.0001)	0.4175 (˂0.0001)
I like my job	0.5350 (˂0.0001)	−0.4577 (˂0.0001)	−0.2565 (˂0.0001)
Good workplace	0.5128 (˂0.0001)	−0.5888 (˂0.0001)	−0.2492 (˂0.0001)

We remarked negative significant correlations between the perception on conflict solving for the patients’ good and the BO score (r=−0.4247, p<0.0001), the safe environment and the BO score (r=−0.477, p < 0.0001), the ineffectiveness when tired and the CS score (r=−0.47, p<0.001), the influence of the excessive workload on the performance and CS score (r=−0.41, p<0.0001), the perception of a pleasant job and the BO score (r=−0.45, p<0.0001), respectively, while the pleasant workplace perception was negatively correlated with burnout (r = −0.5888, p < 0.0001).

Furthermore, we observed the various positive significant correlations: the ineffectiveness when tired and the BO score (r=0.57, p<0.0001), the perception of the excessive workload that could hurt performance and the BO score (r=0.51, p<0.0001), and the STS score (r=0.41, p<0.0001), respectively. Finally, the perception of a pleasant job and workplace positively correlates with the CS score: r=0.53 (p<0.0001), and r=0.51 (p<0.0001), respectively.

## Discussions

Our results indicate that BO varies significantly across the healthcare provider categories and age groups, with younger professionals and certain medical positions experiencing higher levels of burnout. Similar trends have been observed in the international studies, where younger healthcare workers report increased emotional exhaustion due to the workload intensity and lack of experience in stress management [16,17]. The work environment appears to be associated with BO levels, suggesting that workplace conditions may influence stress and emotional exhaustion. The work environment appears to be associated with BO levels, suggesting that workplace conditions may influence stress and emotional exhaustion. The work environment appears to be associated with burnout levels, suggesting that workplace conditions may influence stress and emotional exhaustion.

A study conducted by Crabtree-Nelson et al. investigated the levels of compassion, satisfaction, and BO among nurses in a large Texas healthcare system. The results indicated low levels of compassion and BO, alongside high levels of compassion satisfaction. Factors such as the number of hours worked and specialty influenced these outcomes. Nurses with less than 10 years of experience reported lower levels of CS and higher BO levels compared to those with over 10 years of experience. Additionally, holding a professional certification was associated with higher levels of compassion [18].

CS levels also differ by profession, gender, and age, with women and more experienced professionals reporting higher satisfaction. Studies highlight that experienced professionals develop better coping mechanisms and stronger patient relationships, contributing to the job satisfaction [19,20]. These findings support the potential value of mentorship programs and continuous professional development for CS among younger or less experienced staff members.

STS was found to be more prevalent in certain professional groups, aligning with research suggesting that prolonged exposure to patient trauma can lead to emotional distress [21]. The uneven distribution of the traumatic patient cases among participants may impact the psychometric validity of the STS subscale, reinforcing the need for studies with diverse samples. Additionally, healthcare workers in emergency and intensive care units may be particularly vulnerable, given their frequent exposure to critically ill patients.

The perception of patient safety varied among the healthcare providers, with agreement on a safe working environment, but with concerns regarding the staffing levels and adherence to safety protocols. Similar discrepancies have been identified in the global studies, where inadequate staffing and procedural non-compliance contribute to occupational stress and patient safety risks [22]. Nurses, in particular, expressed concerns over the workload and staffing shortages, findings consistent with the international research, emphasizing the link between nurse-patient ratios and BO [23].

Professional rank and experience influence perceptions of patient safety and well-being. Higher-ranked medical staff (e.g. the specialists) often report greater control over their work and decision-making processes, leading to increased compassion satisfaction and lower burnout levels. In contrast, residents and nurses, often working in high-pressure environments with limited autonomy, may experience heightened stress and BO. These patterns suggest the potential benefit of policies that empower lower-ranked healthcare professionals, such as shared decision-making models and structured support systems. Moreover, younger professionals are more susceptible to work-life imbalances, which can exacerbate stress and burnout. The implementation of flexible scheduling, mental health programs, and peer support initiatives may help mitigate these effects and promote a healthier work environment [24].

In 2022, Yörük et al. conducted a cross-sectional study in Turkey to examine secondary traumatic stress and psychological resilience among healthcare workers during the COVID-19 pandemic. These findings are relevant in the context of the ICU staff resilience explored in our study Factors such as health perception, fear of contagion, and anxiety about infecting family members were identified as predictors of STS. Additionally, the socioeconomic status, health status, age, marital status, professional experience, and the type of work were correlated with the psychological resilience levels. A lower number of resilient workers suffered from STS [25]. The study found associations between the workplace conditions and the professional quality of life.

Safe work environments and effective conflict resolution were associated with lower levels of BO, supporting previous research on the protective effect of the structured support systems in healthcare [26]. Conversely, excessive workload and fatigue strongly correlated with BO and STS, a trend widely documented in the studies on occupational health [27]. While several associations reached statistical significance, their clinical relevance should be interpreted cautiously, as no predefined clinical criteria or thresholds were applied in this study.

CS may reflect stronger professional identity and motivation, contributing to resilience in mitigating stress, especially for the ICU workers [28]. International research confirms that positive job perception can buffer against work-related distress and promote resilience in the healthcare workers [29].

Higher emotional intelligence has been associated with improved stress management and team functioning and with improving the safety climate perception [30]. The healthcare workers in the ICU with higher emotional intelligence are more adept at handling stress, making effective decisions, and fostering team-work, ultimately contributing to both their well-being and patient safety.

A well-organized work environment, with adequate equipment and effective communication, is associated with lower BO and increased job satisfaction. Conversely, fatigue, high workload, and ignoring procedural rules were correlated with decreased CS and increased STS. These findings underscore the potential importance of measures aimed at optimizing working conditions, reducing staff overload, and enhancing teamwork. Effective teamwork, based on communication and problem-solving skills, has been associated with the provision of safe and efficient health services [31]. Studies show that strong team dynamics mitigate the BO effects and improve professional satisfaction. Encouraging interprofessional collaboration and team-building activities can further strengthen the work-place cohesion.

Ongoing training and education may help reduce the clinical risk and improve the safety outcomes. Specific training for anesthesia professionals, for instance, is recommended to ensure optimal care and prevent errors [32]. The constant pursuit of quality values and the promotion of a safety culture are fundamental for continuous improvement in hospitals [33]. Hospitals and healthcare institutions should prioritize structured training programs that emphasize emotional resilience, teamwork, and crisis management. These strategies may be beneficial; however, their effectiveness warrants further investigation.

Regarding STS, only limited differences were observed across professional subgroups. This relative lack of variation may reflect a broadly shared exposure to emotionally demanding clinical situations within ICU settings, irrespective of professional role. The modest subgroup differences suggest that STS may be more strongly related to overall working conditions and cumulative emotional burden than to specific demographic or professional characteristics. Therefore, these findings should be interpreted cautiously and highlight the need for organizational-level support strategies.

To our knowledge, this is one of the few studies that examines the association between Safety Attitudes Questionnaire (SAQ)–derived safety perceptions and Professional Quality of Life (ProQoL) domains across multiple professional roles in a multicentric intensive care unit setting, providing novel insights into organizational well-being. These findings are consistent with international recommendations emphasizing organizational responsibility for healthcare workers’ mental health and well-being. Both the World Health Organization (WHO, 2022) and the NICE guideline NG212 underline the importance of institutional strategies focused on improving working conditions, promoting psychological safety, and supporting staff well-being in high-risk clinical environments. [33,34].

### Limitations

This study has several limitations that should be acknowledged. The cross-sectional design precludes causal inference between safety climate perceptions and professional quality of life outcomes. A major methodological limitation of this study is the reliance on univariate statistical analyses (ANOVA, Pearson correlations) without the use of multivariable regression models. The absence of multivariable analyses prevented adjustment for potential confounding factors and therefore limits the internal validity of the findings. In particular, heterogeneity in hospital settings and other unmeasured covariates may have influenced the observed associations. Furthermore, we considered an r value >0.4 to indicate clinical relevance in this context, as no universally accepted cut-off is available in the literature. Moreover, we considered a r value above 0.4 to be clinically relevant in this context, since no single cut-off value for this is available in the literature.

Key variables such as age, gender, professional role, and hospital status are likely interrelated, and these mutual associations could not be fully disentangled. Data were collected using self-reported questionnaires, which may introduce reporting bias. A shortened 10-item version of the Safety Attitudes Questionnaire was used, potentially limiting coverage of all safety climate dimensions. Information on professional experience and seniority was not available, restricting further stratified analyses. Finally, the exclusive focus on intensive care unit settings may limit the generalizability of the findings to other healthcare contexts.

## Conclusion

The results indicate associations between workplace conditions and professional quality of life indicators, including BO, CS, and STS in the ICU departments. Strategies such as workload management, fostering teamwork, and ensuring adequate staffing may support improvements in the quality of life of the healthcare professionals. These findings are consistent with the international literature and underscore the potential value of workplace support systems and mental health resources in promoting the ICU staff well-being.

## Supplementary Material

Supplementary Material Details
