# Supplementary material for: Quality of life and patient safety: the impact of the work environment on the well-being of medical staff in ICU settings
Source: J Crit Care Med (Targu Mures). 2026 Jul 27;12(3):444–52. doi: 10.62838/jccm-2026-0025 (PMC13403014; doi:10.62838/jccm-2026-0025)
Supplement: Supplementary file 1 — Supplementary Material Details [file jccm-2026-0025_Supplementary.pdf]

# Supplementary material

## The SAQ questionnaire
## Answer options: never, rarely, sometimes, often, very often

1. The medical staff is sufficient to cope with the number of patients in the anesthesia and intensive care/surgery department
2. The equipment in the anesthesia and intensive care/surgery department is adequate
3. Disagreements/conflicts are resolved appropriately (i.e. not about who is right, but what is best for the patient)
4. Doctors and nurses in the anesthesia and intensive care/surgery department work together as a team
5. I would feel perfectly safe if I were treated here in the anesthesia and intensive care/surgery department
6. Staff in the anesthesia and intensive care/surgery department frequently ignore rules or instructions
7. I am less effective at work when I am tired
8. When my workload becomes excessive, my performance is affected
9. I enjoy my job
10. This anesthesia and intensive care/surgery department is a good place to work

## Questionnaire (PROQ 5)
## Answer options: never, rarely, sometimes, often, very often

1. I am happy.
2. I am concerned about helping more people.
3. I get satisfaction from being able to help people.
4. I feel connected to others.
5. I startle or am surprised by unexpected sounds.
6. I feel refreshed after work.
7. I find it difficult to separate my personal life from my professional life.
8. I am not as productive at work because I lose sleep because of the traumatic experiences of the people I care for.
9. I think I may have been affected by the traumatic stress of the people I care for.
10. I feel captivated by my job.
11. My work has made me feel “on edge” about things.
12. I enjoy my work as a healthcare professional.
13. I feel depressed because of the traumatic experiences of those I care for.
14. I feel as if I am experiencing the trauma of the person I care for.
15. I have beliefs that balance me.
16. I am satisfied with the way I am able to keep up with patient care methods.
17. I am the person I have always wanted to be.
18. My work makes me feel satisfied.
19. I feel tired because of my work as a healthcare professional.

20. I have optimistic thoughts and feelings about those I care for and how I can help them.
21. I feel overwhelmed by my workload.
22. I believe that the way I practice my profession as a healthcare professional makes a difference in the life of the patient I care for.
23. I avoid certain activities or situations that remind me of frightening experiences of the patients I care for.
24. I am proud of what I can do to help patients.
25. After work, I have intrusive, frightening thoughts.
26. I feel “hindered” by the system in practicing my profession.
27. I consider myself a successful healthcare professional.
28. I do not remember important parts of my work when I worked with people who have suffered trauma.
29. As a healthcare professional, I am a very caring person.
30. I am happy that I chose to do this profession

Compassion satisfaction items: 3,6,12,16,18,20,22,24,27,30
Burnout items: 1,4,8,10,15,17, 19, 21, 26, 29
Secondary traumatic stress items: 2,5,7,9,11,13,14,23,25,28
